# Mathematical Optimization Strategy for Effectiveness Profile Estimation in Two-Dose Vaccines and Its Use in Designing Improved Vaccination Strategies Focused on Pandemic Containment

**DOI:** 10.3390/vaccines12010081

**Published:** 2024-01-12

**Authors:** Óscar A. González-Sánchez, Daniel Zaldívar, Erik Cuevas, L. Javier González-Ortiz

**Affiliations:** 1 Division of Technologies for the Cyber-Human Integration, University Center of Exact Sciences and Engineering (CUCEI), University of Guadalajara, Marcelino García Barragán 1421, Col. Olímpica, Guadalajara CP 44430, Jalisco, Mexico; daniel.zaldivar@academicos.udg.mx (D.Z.); erik.cuevas@academicos.udg.mx (E.C.); 2 Department of Chemistry, University Center of Exact Sciences and Engineering (CUCEI), University of Guadalajara, Marcelino García Barragán 1421, Col. Olímpica, Guadalajara CP 44430, Jalisco, Mexico; luisj.gonzalezo@academicos.udg.mx

**Keywords:** vaccine optimization, vaccine efficacy profile, COVID-19, numerical deconvolution, national COVID-19 databases, multidimensional fitting, metaheuristic optimization

## Abstract

Since late 2019, most efforts to control the COVID-19 pandemic have focused on developing vaccines. By mid-2020, some vaccines fulfilled international regulations for their application. However, these vaccines have shown a decline in effectiveness several weeks after the last dose, highlighting the need to optimize vaccine administration due to supply chain limitations. While methods exist to prioritize population groups for vaccination, there is a lack of research on how to optimally define the time between doses when two-dose vaccines are administrated to such groups. Under such conditions, modeling the real effect of each vaccine on the population is critical. Even though several efforts have been made to characterize vaccine effectiveness profiles, none of these initiatives enable characterization of the individual effect of each dose. Thus, this paper presents a novel methodology for estimating the vaccine effectiveness profile. It addresses the vaccine characterization problem by considering a deconvolution of relevant data profiles, treating them as an optimization process. The results of this approach enabled the independent estimation of the effectiveness profiles for the first and second vaccine doses and their use to find sweet spots for designing efficient vaccination strategies. Our methodology can enable a more effective and efficient contemporary response against the COVID-19 pandemic, as well as for any other disease in the future.

## 1. Introduction

Since the emergence of the COVID-19 pandemic in late 2019 [1], public health services have taken a central position in global pandemic control [2], facing a challenge never seen before [3,4]. Simultaneously, many private and public scientific groups have been working to develop safe and effective vaccines to alleviate this health crisis [5]. After a few months, these objectives were met by some research groups, showing successful results in phase 3 clinical trials [6,7,8,9,10,11,12], demonstrating the safety and efficacy of their respective vaccines to international organizations. To date, vaccines have demonstrated their capacity to reduce the spread of the pandemic, hospitalizations, and deaths in real-world conditions. Unfortunately, recent studies have examined some COVID-19 vaccines, reporting a considerable reduction in their effectiveness against contagion only a few months after completion of the vaccination schedule [13,14,15]. In this situation, it would be necessary to administer several vaccines annually to ensure sustainable pandemic control. This challenge is untenable with current vaccine supply chains [16].

In fact, vaccines are still not affordable for many countries to implement wide-access vaccination programs in their populations [17]. International health organizations have identified this equity challenge as crucial for securing sustainable pandemic control [18,19,20]. Although social restrictions have been proposed as an alternative to vaccination programs, showing their capacity to restrict the spread of the pandemic [21], such restrictions usually produce economic repercussions or decrease the quality of life of the population [22,23]. Conversely, vaccines provide protection while requiring little effort from the population, offering a realistic solution for many countries that cannot afford the economic repercussions of mass social isolation.

Thus, strategies have been developed to improve the use of available vaccines while aiming to reduce confirmed cases, hospitalizations, and deaths [24,25,26]. Among these strategies, classifying the population by exposure or vulnerability to severe COVID-19 is the most common approach. Thus, many countries try to ensure the availability of vaccines for the elderly population, medical staff, or those who are immunocompromised or have underlying health conditions [27]. However, because vaccines have limited availability, improvements in prioritization strategies have been insufficient to allow many governments to offer universal access vaccination programs to target such objectives, even in developed countries [19].

However, the strategies mentioned above made several assumptions, including the consideration of non-time-dependent vaccine efficacies [24,25,26]. Unfortunately, several reports have demonstrated that after reaching maximum vaccine effectiveness, this parameter wanes as the time-from-vaccine increases (i.e., the time from the last dose application) [28,29,30,31]. Furthermore, a data-processing strategy that enables estimation of a time-dependent effectiveness smooth profile was recently proposed, characterizing the beneficial effects induced in a population group by its vaccination process [32]. This strategy demonstrates quantitatively that, at least for the considered example case, vaccine effectiveness highly depends on the time-from-vaccine, and its efficacy may last only a few months (4–5 months) [32].

Until now, scientific efforts have mainly focused on obtaining useful information to select a more convenient population group to be vaccinated. However, as will be shown, this work is focused on increasing the beneficial effect obtainable with each administered dose, by studying not who must be vaccinated, but how they must be vaccinated. On this topic, it must be mentioned that the developers of the BNT162b2 vaccine only tested one value for the time between doses (21 days) [33,34,35,36,37,38], using this value in their phase 3 clinical trial [12]. In the case of the AZD1222 vaccine, most of the studies published by developers reported 4 weeks as the time between doses [39,40,41,42,43]. However, another of their studies considered 8 weeks [44], and other three studies classified the recruited subjects on time periods between doses that were as long as 45 weeks [45,46,47]. In addition, some studies for the AZD1222 vaccine showed, at an exploratory level, that the antibody titers [46] and the vaccine efficacy [47] depend on the considered time between doses considered, highlighting the necessity of taking advantage of such dependence in the real context of vaccine shortage. In fact, a wide interval (up to 12 weeks) for the time between doses of the AZD1222 vaccine was considered at least in some countries [48,49]. Nevertheless, the doses were administered four weeks apart in the phase 3 clinical trial of this vaccine [11]. The latter demonstrates that the time between doses is a promising parameter that has been insufficiently studied, as the available studies did not offer a systematic strategy to select a time between doses that adapts to the local requirements, aiming to maximize the impact of each dose administered.

Thus, the complementary approach proposed here can contribute to the aim of reducing the number of vaccines required to protect a population by increasing the protection period in each recipient in whom a minimum objective efficacy is maintained.

The proposed strategy studies the effects on the vaccine’s global efficacy profile, expectably producible by the variation in the number of days between doses on two-dose vaccines. The aim of performing such variations is to maximize the protection time (the time during which the vaccine effectiveness value is higher than a pre-established value) achievable by two-dose vaccination schemes for any pre-established minimal efficacy value. Thus, local public health institutions can design specific vaccination schemes that meet the local efficacy requirements. Such a design may consider local vaccine acceptance, social behaviors, pathogen spread, or other parameters that local authorities consider convenient to handle the disease better.

Accordingly, the inter-relationship of the following three parameters must be considered to estimate the vaccine’s effectiveness in a given population: (a) the beneficial effect profile (B); (b) the vaccine effectiveness profile (E); and (c) the vaccination profile of the population (f). The beneficial effect profile (B) measures the relative incidence of confirmed cases in the study group compared with a reference group, providing a ratio that reflects the vaccine’s overall impact in reducing cases. The vaccine effectiveness profile (E) represents the time profile of protection offered by the vaccine, revealing how its effectiveness evolves over time and indicating any decline. The vaccination profile of the population (f) tracks the number of individuals vaccinated over time, offering a dynamic perspective on vaccination progress through the temporal distribution of inoculations.

From a mathematical point of view, the beneficial effect profile (B) can be modeled as a convolution between the vaccine effectiveness, E, and the vaccination profile of the population, f [32]. As demonstrated in a previous study [32], the traditional approach to determine the effectiveness from the quotient between the relative incidence of the confirmed cases in the vaccinated group and the equivalent relative incidence in the unvaccinated group is a particular case of such a convolution process, which can only be applied when the vaccine effectiveness is not time-dependent. Unfortunately, this condition was not fulfilled in the currently considered case, being required to apply a more complex procedure.

Convolution is a fundamental operation that allows us to characterize how the effectiveness of vaccines (E profile) interacts with the distribution and timing of vaccination efforts (f profile) to produce a reduction in the incidence of confirmed cases (B profile) [32].

The used methodology takes advantage of the fact that the B profile can be obtained from registers that track the incidence of confirmed cases in the study and reference groups, whereas the f profile can be obtained from the temporal distribution of inoculations, which enables estimations of the vaccine effectiveness profile (E) by applying a process named deconvolution. Deconvolution, also known as inverse convolution, is the reverse of the convolution process. Convolution combines the functions f and E to produce a third function, B, whereas deconvolution aims to recover the original element, E, from the result of the convolution process (the output B profile) and the known input profile (f profile).

Nevertheless, it must be considered that deconvolution is highly sensitive to noise (e.g., measurement errors or random fluctuations). Therefore, small changes in the inputs can significantly impact the obtained results. To handle this circumstance, in reference [32], the deconvolution problem involved in obtaining the vaccine effectiveness (E) was addressed by defining continuous and smooth profiles to represent the vaccine effectiveness over time. These profiles offer a general understanding of how the vaccine’s effectiveness evolves after its administration. However, these profiles rely on a specific equation that omits the details that allow the separation of the effect of each vaccination dose, which is valuable information.

Thus, this study approached the deconvolution problem as an optimization process, where the task of recovering the unknown element, E, from the observed output, B, and the input, f, was translated into finding the best approximation of E that minimizes the deviation between the real-world B profile and the estimated one.

To solve this optimization problem, a particle swarm optimization (PSO) algorithm was tailored for this application. PSO is a well-known classic method that can optimize continuous nonlinear functions [50,51,52,53,54]. Its capabilities enable cost functions to be optimized even in highly dimensional spaces such as those considered in this study, where more than 200 parameters must be simultaneously fitted. By leveraging the strengths of PSO, the search space can be explored, looking for the deconvolved function E that best represents the original output, B, and input, f. By adopting this optimization-based approach, the proposed method offers flexible and contoured profiles that provide insights into the effect of each individual dose over time, named “malleable profiles.” These profiles offer a detailed understanding of the temporal dynamics of vaccine effectiveness, enhancing our ability to analyze and interpret the impact of each dose. Another important advantage of this approach is that treating the deconvolution problem as an optimization task can prevent the imposition of restrictions or assumptions on the data. This aspect is significant, as assumptions or restrictions can potentially introduce bias and disturb the results. By adopting a more flexible approach, the users obtain deconvolution results that are driven solely by the data and optimization process, leading to more accurate and reliable outcomes.

The remainder of this paper is organized as follows. Section 2 contains a general overview of the whole method, as well as the required profiles to apply this methodology and an explanation of how such profiles were obtained. Section 3 explains how to employ the profiles detailed in Section 2 to estimate the longest time between doses that holds a minimal desired effectiveness for the maximum time period, and also contains a recommendation on how to select a desirable minimal effectiveness value for a given population. This section also compares the results of this methodology and previously reported methods. Section 4 discusses the findings of this study and how such findings could help control the COVID-19 pandemic or any other future pandemic for which vaccines with two doses showing time-dependent efficacies have been developed. Finally, the conclusions are presented in Section 5.

## 2. Materials and Methods

To estimate the vaccine effectiveness profile and the longest time between doses that achieves a desired minimal effectiveness value, the following sequential process must be performed:

(a) Estimate the effectiveness profile of the global vaccination scheme (two-dose scheme; Section 3.1), employing the vaccination profile of the interest group (Section 2.1) and its real-world beneficial effect profile (Section 2.2).
(b) Estimate the effectiveness profile of each vaccination dose (Section 3.2), considering the vaccination scheme used on the interest group, a general interaction model (Equation (8)), and the aforementioned effectiveness profile of the global vaccination scheme (Section 3.1).
(c) Build a profile indicating the number of days which have elapsed between the application of each dose that offers the most extended period of protection for each possible vaccine effectiveness of interest (Section 3.3), considering the above-mentioned general interaction model (Equation (8)), and the effectiveness profile of each vaccination dose (Section 3.2).
(d) Estimate the profile of the basic reproductive number ($R_{0}$) for the region of interest (Section 3.3.1).
(e) Calculate, considering the real-world range of $R_{0}$, the minimal vaccine effectiveness required to progressively decrease the number of confirmed cases, forcing a convergent response on the system (Section 3.3.1).
(f) Obtain the maximum time between doses that holds at least the desired minimum effectiveness value, calculated in clause e (Section 3.3.1), using the profile obtained in clause c (Section 3.3).

### 2.1. Vaccination Profile

**Figure 1:**
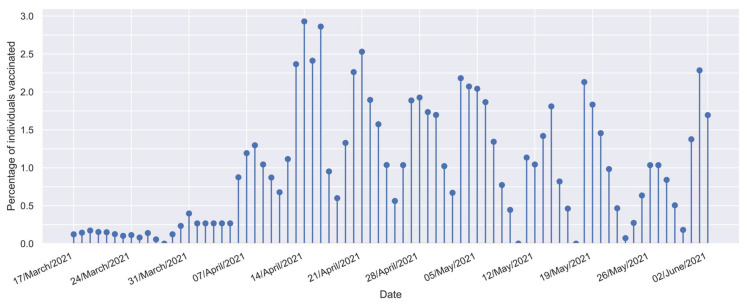
Vaccination profile for Mexican individuals aged 60 years old or more [32].

Vaccination profile data for Mexican individuals aged ≥60 years were collected by the Government of México Health Secretary [55,56,57]. The vaccination profile (f[t]) was composed (for the interest study period) of 78 daily values representing the percentage of individuals vaccinated each day against COVID-19 with any vaccine from the set of approved vaccines in Mexico [58]; the majority of individuals in this group were either vaccinated with two doses of the BNT162b2 (Comirnaty^®^) vaccine [12], or two doses of the AZD1222 (Vaxzevria^®^) vaccine [11]. The vaccination profile considered in this study is shown in Figure 1 [32].

It is important to mention that the data available only enable characterization of the effectiveness profile of the pool of vaccines administrated to Mexicans in the 60+ group. Therefore, for this example, the obtainable results represent the combined behavior of all types of vaccines administrated to the study group.

### 2.2. Beneficial Effect Profile

The beneficial effect profile is a time-dependent profile that shows the increment or reduction in the relative incidence of a disease in a population [32]. This profile is calculated by considering the complement of the ratio of the disease incidence in a study group ($I_{study\;group}\left[t\right]$) relative to a reference group ($I_{reference\;group}\left[t\right]$), as shown in Equation (1) [32].

(1) $$B\left[t\right]=1-\frac{I_{study\;group}\left[t\right]}{I_{reference\;group}\left[t\right]}$$

**Figure 2:**
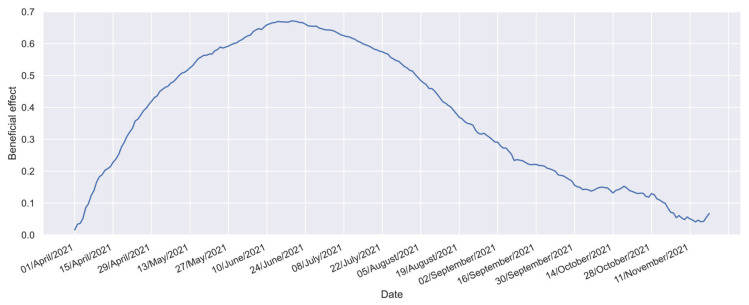
Beneficial effect profile for Mexican individuals aged 60 years or older [32].

In this study, the beneficial effect profile, denoted as (B), is detailed in Figure 2 [32]. This profile considers 232 points calculated from the daily cases reported in the Mexican national COVID-19 database published by the Epidemiology General Direction (DGE, by its initials in Spanish) [59] and population statistics from the Mexican National Institute of Statistics and Geography (INEGI, by its initials in Spanish) [57].

Notably, the B profile reflects the protective effects induced by vaccinated individuals as well as previously infected individuals. However, in the example case (Mexicans in the 60+ group), the total number of previously infected individuals in the study group during the whole study period (175,509 individuals) only represents 1.5% of the vaccinated population (more than 10 million fully vaccinated individuals). Thus, their effect on the B profile can be considered negligible. Nevertheless, a recommendation could be to consider their effect when a higher percentage of the population had been infected (e.g., >15%). Similarly, although the effect of circulating strains could partially influence the B profile, estimation of the effect of each strain in the system is outside the scope of this work.

## 3. Results

Obtaining the proposed profiles followed a sequential procedure, as described in this section. Initially, the global vaccine effectiveness profile was estimated (as detailed in Section 3.1). Subsequently, this profile was used to estimate the effect of each vaccination dose (Section 3.2). Then, an exhaustive analysis of the effect of the time between doses had to be performed to determine the best scenario for obtaining the desired minimum effectiveness value during the longest possible time (Section 3.3). In addition, because the desired effectiveness depends on the local conditions (e.g., the current or foreseeable virus spread level—$R_{o}$ value—and the percentual vaccination coverage—V value—), in Section 3.3.1, the inter-relationship among such variables is studied. Finally, a comparison between the proposed effectiveness profile and the equivalent information published previously is presented in Section 3.4.

### 3.1. Estimation of the Global Vaccine Effectiveness Profile

As previously mentioned, to estimate the global vaccine effectiveness profile, it is required to consider the inter-relationship among the vaccine effectiveness profile (E), the beneficial effect profile (B), and the vaccination profile of the population (f), as expressed in Equation (2). In this case, convolution characterizes how the vaccine’s effectiveness (E) interacts with the distribution and timing of vaccination efforts (f) to induce a reduction in the relative incidence of confirmed cases in the studied population (B profile).

(2) $$B\left[t\right]=\left(f*E\right)\left[t\right]=\sum_{n}f_{v}\left[t-k\right]E\left[k\right]$$

Equation (2) considers that the populational benefits observable on the macroscale (reductions in the relative incidence of confirmed cases; B profile) are composed of small contributions made by each subgroup (individuals vaccinated each day; f profile), weighted by the protection that the vaccines induce k days after each vaccination scheme is completed (the desired E profile); a more detailed description of Equation (2) was presented in a previous paper [32].

However, considering that a physical phenomenon is being studied, the existence of perturbations in the measurements is expected. Therefore, obtaining the desired E profile implies executing the deconvolution of noisy data. As previously mentioned, classical methods to deconvolute signals tend to generate non-realistic solutions in noisy systems (owing to the high relation between variables that produce error accumulation in the system, making it unstable) [60]. Several methods have been developed in the signal processing field to offer more realistic solutions and simultaneously reduce the deviation concerning the experimental data (for signals [61] or images [62]).

For vaccine characterization, a method that handles instabilities by proposing a general function that forces the system to generate smooth profiles that follow the expected global vaccine effectiveness profile shapes has been developed [32]. Although this method offers an outline of the global vaccine effectiveness profile, it is incapable of characterizing the individual effects of each dose. Moreover, as such a profile can generate only one concavity in the estimated function, multimodal systems such as those expected in poly-dose schemes are beyond the capabilities of such a method.

Thus, this study proposes a novel strategy to address these limitations. A particle swarm optimization [50,51,52,53,54] algorithm (PSO) tailored to this application is applied for this strategy. This algorithm considers each daily value of the E profile as a dimension for optimization (232 daily values were considered). In each iteration, this algorithm will propose a batch of candidate E profiles (50 candidates) that are convolved with the available vaccination profile (f), to identify a profile that deviates the least from the real-world beneficial effect profile (B).

Although a comprehensive overview of the PSO algorithm can be found in other sources [54], key aspects of this methodology will be outlined here. The process begins by establishing a group of E profile candidates, which are randomly generated (in this study, 50 candidate profiles were created). Each profile is uniquely identified by the subscript j.

Later, each proposed profile is subsequently modified in each iteration, as indicated in Equation (3):

(3) $$E_{kj}^{i+1}=E_{kj}^{i}+m_{kj}^{i+1}$$

where $E_{kj}^{i}$ (the old value) and $E_{kj}^{i+1}$ (the new value) are the effectiveness values that the jth candidate of the E profile assumes the vaccine will exhibit k days after administration of the second dose. Such values were sequentially assigned in the i and i+1 iterations, respectively. In addition, $m_{kj}^{i+1}$ is the modification that each of such values will undergo in the i+1 iteration.

The modification applicable to each previous candidate value can be calculated considering three terms that must be added, as indicated in Equation (4).

(4) $$m_{kj}^{i+1}=\omega m_{kj}^{i}+c^{i}\left({r1}_{kj}^{i}\left({P_{kj}^{i}-E}_{kj}^{i}\right)\right)+s^{i}\left({r2}_{kj}^{i}\left({S_{k}^{i}-E}_{kj}^{i}\right)\right)$$

The first component of $m_{kj}^{i+1}$ in the PSO algorithm, known as the inertial term, focuses on the prior value of the modification to prevent abrupt changes. The second element, the cognitive term, guides candidate solutions towards their optimal historical performance. This encourages exploration of the search space to identify promising areas. The third, the social term, reflects the influence of the entire population on individual behavior, steering the search towards the best solution found within the group, thus refining the current top solution. Importantly, random numbers (${r1}_{kj}^{i}$ and ${r2}_{kj}^{i}$) influence the cognitive and social terms, meaning that their impact varies throughout the search process. Additionally, the significance of each term is determined by the relative magnitudes of the ω, $c^{i}$, and $s^{i}$ coefficients, with the latter two being adjusted in each iteration. For this study, two decreasing exponential functions were utilized to calculate these coefficients. After updating all effectiveness values, $E_{kj}^{i+1}$, a moving average [63] is applied. This step is essential for achieving smoother profiles at the end of each of the 1000 iterations considered in the process.

The pseudocode for the modified PSO scheme proposed in this study is shown in Algorithm S1, which is included in the Supplementary Materials SM1.

**Figure 3:**
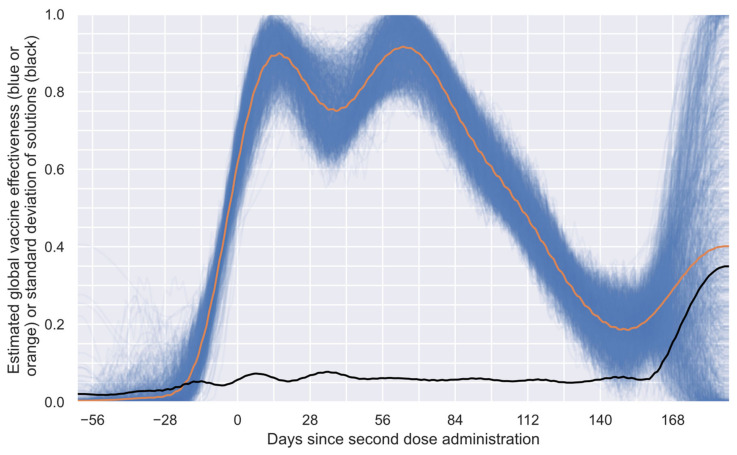
Estimated global vaccine effectiveness profile for the study group and standard deviations of the numerical estimations. One thousand numerical estimations of the E profile (blue curves), the mean solution of all numerical estimations of the E profile (orange curve), and the standard deviation of the numerical estimations (black curve).

In addition, to obtain a more realistic E profile, the methodology was run 1000 times; Figure 3 presents the 1000 obtained numerical estimations (blue curves), the mean solution of such numerical estimations (orange curve), and the standard deviation of the numerical solutions (black curve). In Figure 3, it can be observed that most solutions show agree-shaped profiles on the first segment of the profile (approximately 85% of the considered period), with considerable differences only in the last part. This behavior can easily be explained by considering that the system has only a few subgroups with sufficient time to offer information for the last segment, making the profile inference blurry. This characteristic allows this algorithm to set, in an approximate way, the limit of the estimable profile by considering the available data. To generate the exact limit of the estimable profile, the standard deviation of the solutions (Figure 3, black curve) and an algorithm that detects elbow/knee conditions can be used to determine the date when the system becomes unstable [64].

**Figure 4:**
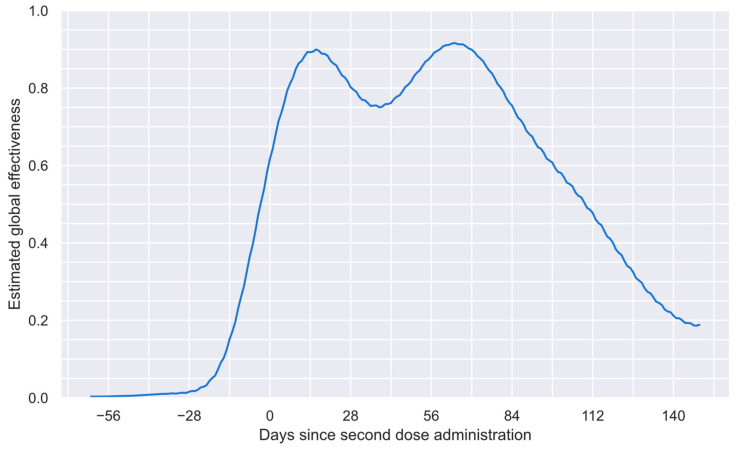
Reliable period of the estimated global vaccine effectiveness profile.

In many engineering problems, it is necessary to determine the point at which a slight change in one variable implies a significant change in another. This problem is commonly known as an elbow or knee condition [64]. Considering the intrinsic characteristics of the numerical estimations performed to obtain the results shown in Figure 3, a knee condition was employed to estimate the last point, after which the system provided unreliable information. Thus, a general-purpose algorithm, known as “kneedle” [64], was used to find such a point. This algorithm was selected because of its simplicity and generality [64]. After applying this algorithm, it was found that 149 days after the second dose was administered, the system offered a blurry solution based on the data considered. Thus, in Figure 4, the reliable period for the estimated global vaccine effectiveness profile is shown. This profile comprises 212 values from 62 days before the second dose to 149 days after such a dose.

After determining the reliable period of the estimated global profile, as shown in Figure 4, the next step is to validate its accuracy against the actual confirmed cases in the study group. To do this, it is helpful to use Equation (1) to solve for $I_{study\;group}\left[t\right]$, as outlined in Equation (5). Furthermore, by considering both Equations (2) and (5), we can formulate Equation (6).

(5) $$I_{study\;group}\left[t\right]=\left(1-B\left[t\right]\right)\left(I_{reference\;group}\left[t\right]\right)$$

(6) $$I_{study\;group}^{calculated}\left[t\right]=\left(1-\left(f*E^{calculated}\right)\left[t\right]\right)\left(I_{reference\;group}\left[t\right]\right)$$

**Figure 5:**
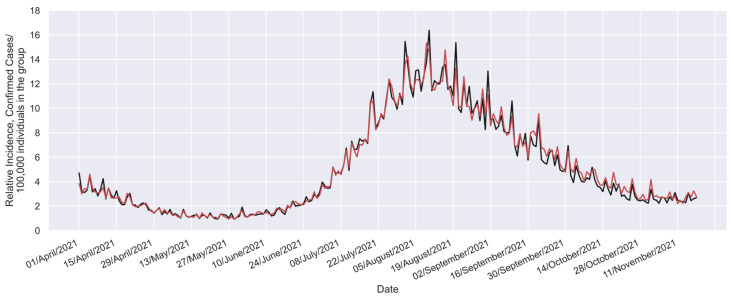
Comparison between the real-world relative incidence ($I_{study\;group}[t]$; black curve) and equivalent relative incidence ($I_{study\;group}^{calculated}\left[t\right]$; red curve) calculated considering the estimated global effectiveness profile (profile shown in Figure 4).

Thus, the real-world data ($I_{study\;group}\left[t\right]$ [57,59]) can be directly compared with the equivalent values ($I_{study\;group}^{calculated}[t]$), which can be calculated considering Equation (6), the available vaccination profile (f profile shown in Figure 1), the $E^{calculated}$ profile (Figure 4), and the incidence in the reference group [57,59]. Such a comparison is shown in Figure 5.

While minor discrepancies are noticeable when comparing the two profiles, these can largely be attributed to the inherent variability and noise characterizing real-world systems. Nonetheless, a clear and consistent overall trend is evident in both profiles. This shared pattern highlights the effectiveness of the proposed methodology in estimating the global vaccine effectiveness profile.

### 3.2. Extracting the Effectiveness Profile of Each Vaccine Dose

Although a global vaccine effectiveness profile is highly desirable for developing successful vaccination programs, knowing the effect of each independent vaccine opens up additional possibilities for surgical adaptation to local necessities. Considering this information, dynamic actions could be taken to address local public health challenges, such as reducing the numbers of confirmed cases, hospitalizations, and deaths, managing the economic impacts and social restrictions, and better protecting susceptible or vulnerable groups such as doctors or the elderly.

Thus, in this study, two basic interaction models were tested to determine the effect of each vaccine dose, which are represented by Equations (7) and (8). Nevertheless, the versatility of the proposed model allows for the consideration of more complex interactions between vaccines that could be used for other vaccines when necessary.

(7) $$E\left(k\right)={1-(1-E}_{1}\left(k\right))\left(1-E_{2}(k+∆k)\right)$$

(8) $$E\left(k\right)=E_{1}\left(k\right)+Q\left(E_{2}\left(k+∆k\right)\right)$$

In Equations (7) and (8), E(k) is the estimated global effectiveness profile (Figure 4) and $E_{1}\left(k\right)$ is an effectiveness profile that represents only the global reduction in confirmed cases that would be induced by administration of the first dose. Notably, since E(k) and $E_{1}\left(k\right)$ start their respective effects at the same time, in the used notation, both parameters must be expressed as a function of the same time scale (k). Additionally, $E_{2}\left(k+∆k\right)$ is an effectiveness profile representing only the global beneficial effect induced in recipients by the second dose. Since such an effect starts later, specifically ∆k days later, in the adopted notation, the functionality with time must be expressed as k+∆k. Note that ∆k is a fitting parameter that can coincide, or not, with the considered time between doses, as these curves are estimated from the induced effect perspective.

The best fit to the profile shown in Figure 4 was obtained when the interaction model represented by Equation (8) was used (additional details are presented in Supplementary Materials SM2). Therefore, such an interaction model was the only one considered in the process of obtaining the optimal time between doses. Nevertheless, further details about the fitting obtained with the other interaction model (in the two proposed versions) have been included in Supplementary Materials SM3.

As many numerical profiles can satisfy the constraints of the proposed model, a smooth continuous equation that represents the time dependence of the effectiveness of each dose must be considered. Thus, a recently proposed equation for estimating the global effectiveness profile [32] was considered in this study (Equation (9)). However, such an equation is now used to fit the effectiveness of each independent dose instead of considering it for the global effectiveness profile. This selection was made considering the high malleability of the family of profiles that can be generated with this equation. Thus, Supplementary Materials SM4 presents two illustrative videos to appreciate such a malleability. There, an ordered sweep of each parameter is performed in the first video. In contrast, in the second video, a simultaneous random exploration of the eight parameters of such an equation is conducted. Thus, in the considered decoupling process, eighteen parameters must be fitted.

(9) $$E_{1,\;or\;2}\left[k\right]=\left(\left(A_{L}\right)\left(\frac{e^{\left(k+D_{L}\right)\left(C_{L}\right)}-e^{-\left(k+D_{L}\right)\left(C_{L}\right)}}{e^{\left(k+D_{L}\right)\left(C_{L}\right)}+e^{-\left(k+D_{L}\right)\left(C_{L}\right)}}\right)+B_{L}\right)\left(\left(A_{R}\right)\left(\frac{e^{\left(k+D_{R}\right)\left(C_{R}\right)}-e^{-\left(k+D_{R}\right)\left(C_{R}\right)}}{e^{\left(k+D_{R}\right)\left(C_{R}\right)}+e^{-\left(k+D_{R}\right)\left(C_{R}\right)}}\right)+B_{R}\right)$$

Here, a general optimization algorithm must fit both profiles ($E_{1}(k)$ and $E_{2}(k+∆k$)) to the data of the global vaccine effectiveness profile shown in Figure 4 (E(k)). This study used an algorithm known as GRG Nonlinear [65,66,67,68], which is available on the Microsoft Excel solver tool from Office 365. The recommended parameters and restrictions used for this optimization are shown in Table SM5-1 of Supplementary Materials SM5. The mean square deviation was used as the element to be minimized (details of the fitting process can be analyzed on the spreadsheet used, which is included in Supplementary Materials SM2, jointly with the information presented in the Supplementary Materials included in Ref. [32]).

**Figure 6:**
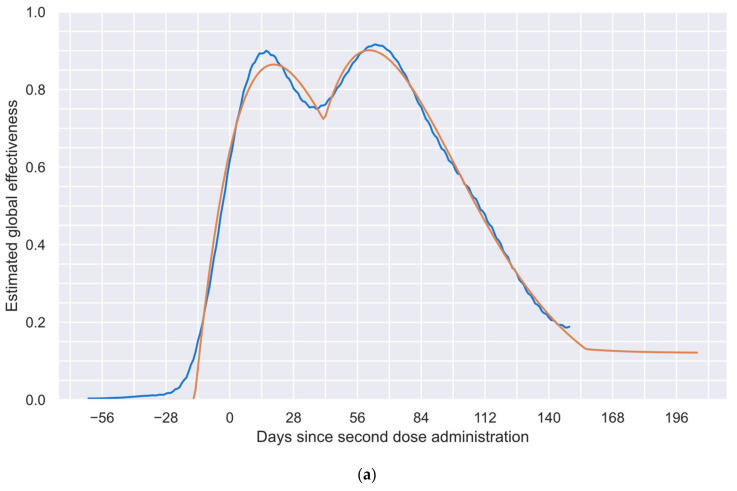

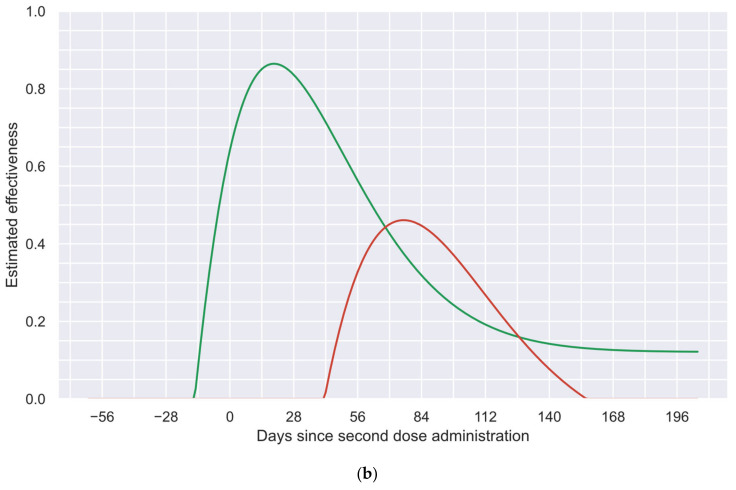
Estimated vaccine effectiveness. (**a**) Original global vaccine effectiveness (blue curve) and fitted global vaccine effectiveness (orange curve). (**b**) Estimated first-dose (green curve) or second-dose (red curve) effectiveness.

In Figure 6, the results of the procedure described above are shown. In Figure 6a, the blue curve represents the previously determined global vaccine effectiveness (reproduced from Figure 4), and the orange curve represents the fitted profile, composed of the independent effect of each of the two doses; the mean square deviation between such profiles is $6\times 10^{-4}$ square units, which denotes a very good fit for a real-world phenomenon. In addition, Figure 6b shows the estimated vaccine effectiveness profile of each dose. Importantly, these profiles were inferred from the induced effect perspective. Therefore, as previously mentioned, the time elapsed between the start of the effects of each dose may not be the same as the time between the administration of both doses.

In Figure 6a, three different periods in the fitted global effectiveness profile (orange curve) can be distinguished: (a) the first one, where only the first dose induces an effect; (b) the second one, where both doses collaborate to increase the effectiveness; and (c) the third one, where only the long-term effects remain.

In the first period, as the second dose has not been applied or has not induced a perceptible effect (see Figure 6b for details), only the first dose is acting. This period is responsible for the first local maximum and the first waning, as the second dose does not start its effect until day 42.

In the second period (from day 42 until day 156), the effects of the second dose not only compensate for the waning of the effectiveness of the first dose (which would have kept decreasing without the second dose), but its rapid increase allows for an increment in the overall global effectiveness profile. This period is responsible for the change in direction after the first local minimum and the generation of the second local maximum observable in Figure 6a. In addition, Figure 6b shows how the effects of both doses act during this period.

In the last period (after day 156), only the long-term effects are preserved, which are observable as a plateau at the end of the global effectiveness profile, as shown in Figure 6a.

Thus, the contoured shape of the fitted global effectiveness profile presented in Figure 6a demonstrates the capabilities achievable by this methodology, allowing for these interaction phenomena to be appreciated. This level of analysis is not achievable by traditional phase 3 clinical trial methodologies, which only allow for the estimation of “wide regions” instead of contoured precise profiles (as will be discussed later).

Finally, it is important to consider that the tailored PSO trends to generate contoured profiles, as shown in the blue curve of Figure 6a. In counterpart, the method employed to independently estimate the effectiveness of each dose tends to estimate sharp changes attributed to each dose (as the green and red curves in Figure 6b). Considering the inherent behaviors of both methodologies, the existence of certain limitations near to the first dose administration could be anticipated (day-31). As a consequence of such a limitation, small effectiveness values (<1%) are induced in the blue curve in Figure 6a, before the application of the first dose. Nevertheless, it was numerically corroborated that their effects were practically imperceptible on the results of the following steps.

### 3.3. Recommended Time between Doses for a Given Effectiveness Value

As mentioned in the Introduction, the time between doses is a promising parameter that enables leverage of each couple of doses in a better way [46,47]. However, in the case of COVID-19 vaccines, to date, such an opportunity has not been explored practically. Considering the few clinical trials performed to test different times between doses, the different scenarios that countries face, and the diverse local pandemic contention strategies implemented, it is logical to expect that the same time between doses will not be optimal in every situation.

**Figure 7:**
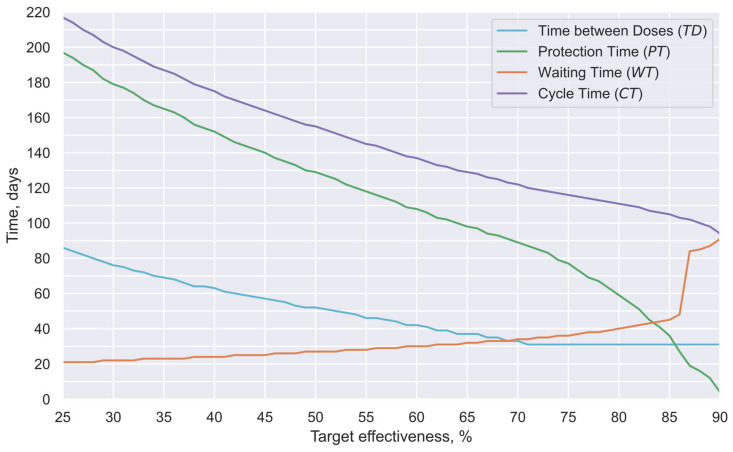
Pareto frontier with the results of the optimization. Recommended time between doses for each effectiveness (TD, cyan curve), the period of time under protection (PT, green curve), the number of days between the first dose and first day under the target protection (WT, orange curve), and the number of days between the first dose and last day under the target protection (CT, purple curve).

Thus, this study estimated the time between doses that could offer the most extended protection period for a given minimal desirable effectiveness; the results of such estimations are presented in Figure 7. Such analysis was performed by considering the possible combinations of times between doses and minimal effectiveness targets. Thus, the proposed basic interaction model (Equation (8)) and the previously characterized vaccine effectiveness profiles for each of the two doses were considered (Figure 6b).

Notably, in a previous study [32], the estimated number of days prior to the application of the second dose during which the first dose effects were perceptible in the group studied here ($t_{fd}$ value) was 31 days. Thus, it could be expected that the $t_{fd}$ value approximately represents the mean time between doses used in the studied group. Therefore, in this study, the optimal time between doses was searched considering only times longer than 31 days. Such a value is slightly longer than the respective times between doses used by manufacturers in their respective phase 3 clinical trials [11,12]. This approach is based on the assumption that prolonging the interval between doses is likely to maintain or reduce adverse effects, rather than increase them.

**Figure 8:**
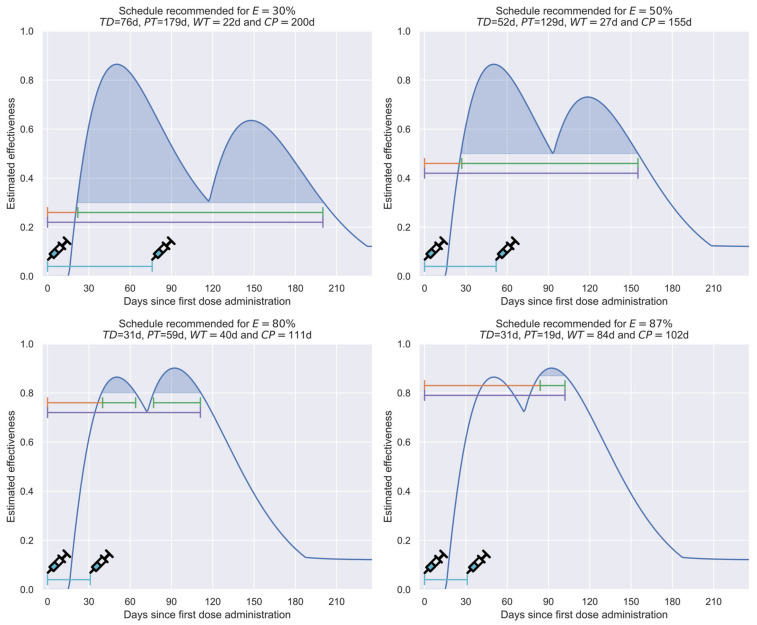
Comparison of the best schedules found in the optimization for 30%, 50%, 80%, and 87% minimum effectiveness values. TD, PT, WT, and CT are graphically included on each plot as blue, green, orange, and purple horizontal lines, respectively. The vaccination days are also graphically indicated with a syringe icon.

Thus, in Figure 7, the cyan curve represents the number of days between doses that offer the most extended protection period for each given effectiveness (time between doses: TD). The green curve shows the number of days in which the obtained effectiveness is estimated to be greater than the target effectiveness (protection time: PT). The orange curve shows the time between the first vaccine application and the first day under the target protection (waiting time: WT). Finally, the purple curve shows the number of days between the first dose application and the last day under the target protection (cycle time: CT). Complementarily, in Figure 8, an illustrative comparison of the effectiveness evolution in four recommended scenarios for the target minimum effectiveness values of 30%, 50%, 80%, or 87% is presented.

Although selecting priority groups to be vaccinated is a strategy that promotes the efficient use of available vaccines, selecting how these groups will be vaccinated also offers a window of opportunity that can have significant impacts in multiple scenarios. Thus, Figure 7 shows how the recommended time between doses decreases as the minimum target efficacy increases (cyan curve). In fact, for objectives more demanding than 70% effectiveness, it is necessary to limit this reduction to the minimum predefined time between doses (31 days), which, as previously mentioned, was selected to eliminate the risk of increasing adverse effects. Consequently, cycle times (purple curve) in these scenarios are comparatively short (<120 days for a 70% minimal effectiveness target vs. 200 days for a 30% target).

Nevertheless, it is even more important that the times under protection (PT, green curve) present a quasilinear relationship in the first segment of the curve. However, in the last section (>70% effectiveness), this curve presents a noticeable decrement in its slope, indicating that it is necessary to sacrifice the length of the period under protection to achieve the most demanding effectiveness values. In scenarios of vaccine shortages, such as those existing during the COVID-19 pandemic, such a phenomenon is highly inconvenient.

Finally, it is convenient to indicate that the waiting time (WT, orange curve) presents a quasilinear behavior, with relatively low values during the first segment of the curve (21–45 days). However, when the target effectiveness is greater than 85%, an abrupt increase occurs in the ordinates of the curve (e.g., 84 days for an objective of 87% effectiveness).

To exemplify, in Figure 8, a comparison among the recommended schemes for effectiveness values of 80% or 87% can be performed. There, while in the first scenario, the waiting time is relatively short and the protection is of medium duration (WT = 40 days and PT = 59 days), in the second scenario, the waiting time is more prolonged, and the protection time is notably shorter (WT = 84 days and PT = 19 days), since the value of the first cusp is lower than the target efficacy.

In addition, in Figure 8, it can be noted that the time elapsed between doses induces a notorious effect in the shape of the efficacy profile obtained, generating systems with long cycle times, but low target effectiveness (schedule recommended for E = 30%); systems that balance duration and the target effectiveness value (schedule recommended for E = 50%); or systems that sacrifice duration in order to achieve high target effectiveness (schedule recommended for E = 80% or E = 87%).

Finally, it is convenient to indicate that the target effectiveness value is the minimum value that the effectiveness profile will have throughout the protection time. For example, of the 179 days in which the recommended profile for E = 30% will offer protection >30%, only three regions will present values close to 30% (less than ten days); most of the time, such a profile will offer significantly higher values, having an average effectiveness of 57%, and even reaching maximum effectiveness values of above 80%. Thus, the profiles generated employing this strategy will allow rapid convergence, since the average effectiveness will be, in most cases, notoriously higher than the threshold effectiveness.

#### 3.3.1. Basic Reproduction Number, $R_{0}$

In epidemiological vigilance, one of the most significant parameters to quantify the social dissemination rate of a disease is the basic reproduction number, usually known as $R_{0}$ [69]. This parameter is typically defined using Equation (10), where β represents the number of individuals infected by a disease in a given period and γ represents the number of individuals recovered in the same period.

(10) $$R_{0}=\frac{\beta }{\gamma }$$

Practically, this parameter estimates the average number of infected individuals directly contaminated by a single case. Thus, if the $R_{0}$ parameter is smaller than one, the number of active confirmed cases will decrease (as more persons recover from the disease than those becoming infected). However, if this value exceeds one, the number of active confirmed cases will progressively increase.

For the example case, considering a recovery period of 14 days, the $R_{0}$ parameter can be estimated by dividing the number of active cases contaminated during such a period (CC(t+14)) by the corresponding number of original active cases (CC(t)), as shown in Equation (11).

(11) $$R_{0}\left(t\right)=\frac{CC\left(t+14\right)}{CC\left(t\right)}$$

**Figure 9:**
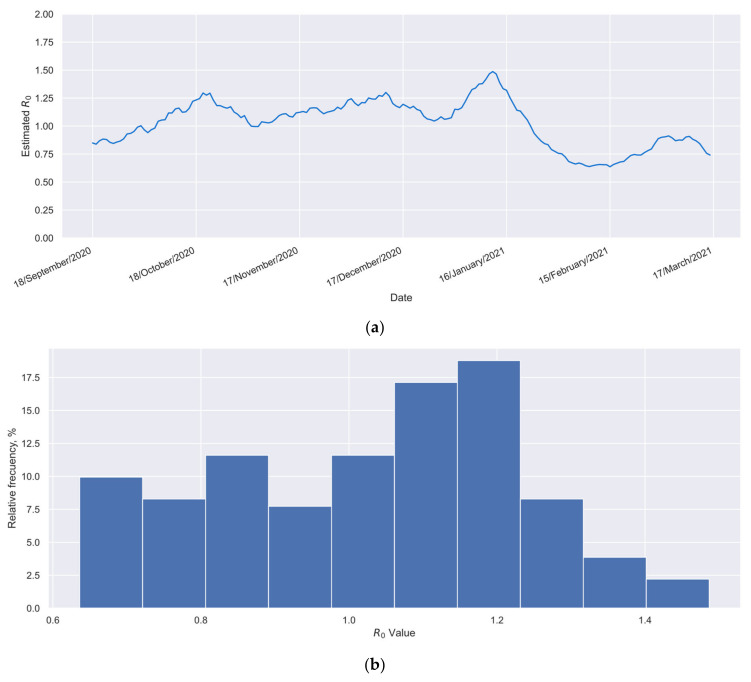
Information about the basic reproduction number ($R_{0}$) for the reference group. (**a**) $R_{0}$ time series and (**b**) histogram for the $R_{0}$ values showed by the reference group.

In the present case, the data required in Equation (11) can be obtained from daily cases reported in the Mexican national COVID-19 database published by the Epidemiology General Direction (DGE, by its initials in Spanish) [59]. For a worst-case scenario analysis, Figure 9a presents the time series of the $R_{0}$ index for the reference group over a six-month period. During this time, the group remained unvaccinated, and only a minimal level of vaccination coverage had been achieved in the Mexican population. These data were calculated using Equation (11). Additionally, Figure 9b shows a histogram built with such values. From the information presented in Figure 9, the average $R_{0}$ value for the reference group can be obtained ($\bar{R_{0}}=1.03$), as could its range, which was 0.64 to 1.49.

Among other applications, one of the benefits of knowing the value of $R_{0}$ for a specific population is that, when the percentage of vaccinated individuals in such a population (V) is available, Equation (12) [70] enables calculation of the minimum value of effectiveness (E) required to decrease the value of $R_{0}$ to values smaller than one, which promotes a progressive reduction in confirmed cases (additional details about the obtainment of Equation (12) are available in Supplementary Materials SM6).

(12) $$E>\frac{1}{V}-\frac{1}{R_{0}V}$$

Equation (12) assumes the fulfillment of the following conditions [70]: (a) individuals in the study population mix homogeneously; (b) the distribution of individuals protected through vaccination (and, where relevant, previous infection) is uniform; and (c) all unprotected individuals are fully susceptible. Traditionally, Equation (12) is used for time-independent effectiveness values, but it also applies to time-dependent parameters, as in this study. In the latter case, the E parameter represents the instantaneous E value, measured k days after the second dose administration. Thus, when the instantaneous E value is higher than the value calculated with the right member of Equation (12), the convergence of the confirmed cases is forced in the system, and the number of active cases progressively decreases in the considered population group. In one previous study [70], the behavior of Equation (12) was widely analyzed, considering traditional systems where the effectiveness is not time-dependent. There, it was demonstrated that high vaccination coverages (e.g., V≥70%) and high effectiveness values (e.g., E≥80%) must be achieved to force the convergence on systems that show high $R_{0}$ values (e.g., $R_{0}\geq 2.5$). The $R_{0}$ value in a locality reflects the balance between the infectiousness of the prevalent variant and the effectiveness of both pharmacological and non-pharmacological protective measures in place. This emphasizes the importance of implementing such protective actions to prevent high $R_{0}$ values, which can be challenging to manage solely through vaccination in real-world conditions.

Thus, Equation (12) allows us to determine the recommendable minimum effectiveness value required to produce a progressive reduction in confirmed cases in the considered location. For the example case (elderly Mexicans during the period September 2020 to April 2021), considering that 95% of the historical values of $R_{0}$ were lower than 1.33 (Figure 9), and 78% of the interest group became vaccinated (Figure 1), an E value of at least 32% could be considered appropriate, as shown by Equation (13). Therefore, considering such effectiveness and the profiles of Figure 7, a time of 73 days between doses could be considered appropriate for the example case.

(13) $$E>\frac{1}{0.78}-\frac{1}{\left(1.33\right)\left(0.78\right)}≅0.32∴E\geq 32\%$$

In this case, the more prolonged separation between doses (73 days instead of 31 days) would produce a significant increment in the protective time, increasing the protection time from a few more than 19 weeks with the schedule administrated (TD=31 days; Figure 8), to practically 25 weeks with the new vaccination schedule (TD=73 days; green curve in Figure 7). Thus, adequate implementation of the proposed schedule would yield, with the same doses, protection to more than 25% additional individuals in a similar way, which is a relevant achievement in shortage scenarios.

Additionally, it is relevant to note that in this calculation, the recursive effect described as follows occurs. Since the new schedule allows a portion of the doses to be used to vaccinate new individuals, the vaccination coverage will increase, and the value calculated by substituting the new values into the right side of Equation (12) will decrease. Thus, a lower effectiveness limit value will be required in the new conditions, which implies that the protection time in the new conditions will increase. Consequently, a certain number of doses can be used in new individuals, which again generates the previously described effect. Thus, as a final result of this recursive effect, the increment in the time between doses will generate an even more significant beneficial effect than that calculated when Equation (12) is only applied the first time.

Notably, in systems showing time-dependent effectiveness values, the E parameter in Equation (12) is now considered the inferior threshold of effectiveness that must be fulfilled, instead of the unique effectiveness value that characterizes a non-time-dependent system. Therefore, the current restriction is considerably stricter than those considered in systems with time-independent effectiveness; for the majority of the protection time, the effectiveness will be noticeably higher than the minimal required threshold, and only in comparatively few days will it be near to the minimal value. Therefore, it could be expected that a more accelerated convergence process than in the traditional systems (time-independent) can be achieved in the current case.

Nevertheless, it must be noted that prior to applying Equation (12) to a specific case, the following non-trivial caveat must be considered: the $R_{0}$ may change sharply with changes in social behaviors or the emergence of new variants that could be more infectious. Thus, it must be highlighted that the $R_{0}$ value used in Equation (13) is only valid for the local social behaviors and the variants that circulated during the studied period among elderly Mexicans. On this matter, international evaluations have identified high heterogeneity in the $R_{0}$ value, even within a country [71]. However, mean estimations range from 2.9 for the ancestral strain [71] to 9.5 for the Omicron variant [72]. Therefore, to anticipate possible changes in social behaviors or variants after vaccination is applied, a generous protection margin should be considered for the $R_{0}$ value to be used in Equation (12).

As previously mentioned, a broad analysis of the behavior of Equation (12) can be found in [70]. However, to evidence the real achievements that can be obtained when only the vaccination is used as a protective strategy, Table 1 considers the relationship among the parameters included in such formulae. Thus, Table 1 presents the minimum effectiveness required to induce a convergent behavior in the local evolution of the pandemic (reduction in the locally confirmed cases), corresponding to some representative combinations of values of $R_{0}$, and vaccination coverages in the population (V).

Table 1 shows that for all tested V values, the required minimum effectiveness (E) sharply increases as the $R_{0}$ value increases. However, such an increment is less sharp when the vaccination coverage is high. It is important to note that even with moderate vaccination coverage levels, such as 60% or 70%, there are scenarios where vaccination efforts alone are insufficient to achieve convergence. Furthermore, in certain cases, particularly when $R_{0}$ exceeds 5, even high vaccination coverage (≥80%) may demand exceptionally high vaccine effectiveness (E≥90%) to facilitate convergence.

Lastly, it is crucial to understand that the protection times (PT) are strongly determined by the required minimum effectiveness (E), with PT decreasing as E increases. Such a decrease becomes even more pronounced when E surpasses 70%. Thus, as previously mentioned, when the E and V values are limited, convergence can hardly be achieved in systems showing high $R_{0}$ values. Furthermore, when the required E values are comparatively high, the afforded duration of the protection only lasts some weeks.

### 3.4. Comparison against Previously Available Information

**Figure 10:**
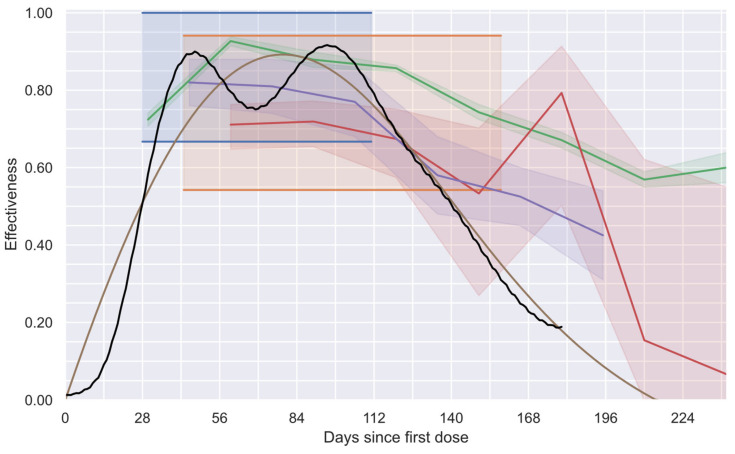
Effectiveness profiles estimated by considering different methodologies. The 95% CI reported in phase 3 clinical trials for AZD1222 [11] (in orange) and BNT162b2 [12] (in blue) vaccines. Time-dependent profiles reported by Tartof [13] (in purple), Chematelly [14] (in red), and Lin [15] (in green) for the BNT162b2 vaccine. Profile estimated with a “smooth deconvolution” proposed by González-Sánchez [32] (brown curve). Profile estimated using numerical deconvolution and a tailored PSO optimization (black curve).

Concerning the effectiveness profiles, in Figure 10, the proposed global vaccine effectiveness profile (black line) and the profiles obtained with three alternative methodologies previously proposed are shown. The first alternative methodology is the one used in phase 3 clinical trials performed by the manufacturers of the vaccines administrated to most of the individuals in the studied group (BNT162b2 [12] and AZD1222 [11] vaccines). Thus, the 95% confidence intervals (95% CI), the only values each manufacturer offers for their study groups, which were assumed to be valid during the complete study, are presented in Figure 10 as shadowed regions in blue and orange, respectively. The second alternative considers the equivalent results of three independent studies [13,14,15] that estimated the evolution of the effectiveness of the BNT162b2 vaccine over time, reporting monthly values of effectiveness with their respective 95% CI. To estimate such values, the individuals had to be classified by their time from vaccination (usually, monthly groups were considered). To better appreciate their results, in Figure 10, a line connecting their effectiveness values and a shadowed region to observe their connected 95% CI were added (purple [13], red [14], and green [15]). Finally, the third alternative to be compared considers the profile obtained using a deconvolution process that forces the system to generate smooth profiles with eight degrees of freedom (“smooth deconvolution” [32]). The obtained continuous profile and its respective 95% CI are shown in brown. It is important to note that such a study and the current study (black line) had almost imperceptible 95% CI due to the colossal number of individuals considered in these studies (more than 15 million in the study group and more that 18 million in the reference group).

Qualitative agreement between all studies during the first five months after the first dose administration can be observed in Figure 10. The observed minor discrepancies can be attributable, among other reasons, to the slight differences among the groups considered. Note that in references [14,32], the group 60+ (individuals older than 60 years) was considered, while in other studies [11,12,13,15], groups 65+ were considered. In addition, in the current study and in reference [32], a group vaccinated with more than one type of vaccine was considered, whereas in the other studies, only the BNT162b2 vaccine was used (except in reference [11], which considered the AZD1222 vaccine). On the other hand, this study’s results and those reported in studies considering the time evolution of the vaccine effectiveness [13,14,15,32] demonstrate a progressive decrement in such a parameter after a maximum value is reached, corroborating the waning of the vaccine effectiveness after a few months of having administrated the first dose.

Complementarily, a quantitative comparison of the proposed profile with the other options confirms that: (a) the current profile provides a more precise description of the vaccine effectiveness compared with the original single value offered by the vaccine manufacturers (blue [12] and orange [11]); (b) the length of the 95% CI obtained with the current strategy is negligible compared with those obtained with some of the other equivalent methodologies (purple [13], red [14], and green [15]); (c) since the current methodology does not require the consideration of confirmed cases with different times from vaccination as if they were equivalent, the involved bias is eliminated; and (d) although the smooth profile proposed by Gonzalez-Sanchez (brown [32]) accomplishes the aforementioned benefits, the low number of degrees of freedom used only allowed the generation of smooth profiles that are incapable of describing the real behavior of two-dose vaccination schemes with enough fidelity, preventing the characterization of the individual effects of each dose, which can be achieved with the proposed methodology.

Thus, the proposed methodology is the best available option for generating global effectiveness profiles for two-dose schemes. Moreover, to the best of our knowledge, this is the only study that has generated profiles that can be quickly processed to extract the effectiveness of each vaccine individually.

## 4. Discussion

The global health quandary generated by the COVID-19 pandemic and its persistence for more than three years has implied many challenges not seen in other pandemics [3,4,22,23]. Global interconnections and the international transit of goods and persons have generated a pandemic in which all countries depend on each other to control their infections [73]. Considering this panorama, the international availability of vaccines could be viewed as a primary public health requirement for the pandemic contention [27].

Regarding vaccine availability, the limited production capacity of this resource [17,19] has motivated the development of strategies to use available vaccines efficiently. The strategies range from vaccinating the most vulnerable groups (e.g., the elderly) or the most exposed individuals (e.g., doctors), up to prioritization of potential super-spreaders (e.g., teachers and public transport drivers). Additionally, several research groups have developed models to study different strategies for prioritizing vaccines, aiming to reduce the numbers of confirmed cases, hospitalizations, and deaths [24,25,26]. However, many of these strategies consider unrealistic assumptions, including the consideration of a time-independent vaccine effectiveness value. This assumption implies non-negligible deviations, which could lead to the design of ineffective public health strategies.

The traditional vaccine efficacy estimation method assumes the use of vaccines with time-independent efficacies, i.e., vaccines that induce an immunological response preserved for extensive periods. Nevertheless, this assumption has been proven wrong for at least some COVID-19 vaccines, which demonstrate effectiveness with strong waning as the time from the last dose increases [28,29,30,31,74]. Concerning this methodological bias, several research groups have studied the evolution of COVID-19 vaccine effectiveness by considering monthly values instead of a single value to represent the effectiveness of each vaccine type [13,14,15]. However, the limited number of confirmed cases considered in these studies generates biased and imprecise profiles, preventing its use in successful public health strategies. In a previous study [32], the limitations characterizing these methodologies were deeply analyzed, mentioning that, to surpass such limitations, it is required to recruit at least 1000 times more individuals than in traditional phase 3 clinical trials (for the current case, more than 33 million of individuals were considered), which is an impractical challenge. Fortunately, national databases represent a suitable alternative that can be used, as in this case, when vaccines have been broadly distributed.

Thus, the proposed novel approach uses information grouped in national databases containing data from millions of individuals. The availability of information concerning millions of individuals improves the resolution of the effectiveness profile without reducing statistical robustness. In addition, the proposed method solves the limitations of its predecessor method [32] by stabilizing a system with more degrees of freedom (212 instead of 8). The noticeable increment in the number of degrees of freedom and the consideration of a general interaction model gives the generated profile enough flexibility to be split into two independent effectiveness profiles (one for each dose), which is impossible to achieve with the precedent procedure [32].

The split of the respective effectiveness profiles (one for each dose) opened the possibility of evaluating the effect of increasing the time between doses proposed by pharmaceutical companies, looking to enlarge the protection time of the complete vaccination scheme but maintaining effectiveness levels higher than a pre-established threshold value. Although, in this study, such a threshold was defined to promote local convergence to zero on the number of confirmed cases, taking advantage of the fact that this threshold could be calculated considering the current conditions in the considered locality (the basic reproduction number—$R_{0}$— and the vaccination coverage—V—), this methodology can also be adapted to achieve other aims (e.g., finding a sweet spot between the protection time and minimum effectiveness value, or looking for a high effectiveness value during local celebrations). Thus, the schedules recommended in this study aim to maintain a desired minimum effectiveness for the longest possible time. Therefore, this methodology could reduce the number of doses required to protect a given population.

Thus, the availability of a method to vary the desired effectiveness facilitates the design of surgical public health strategies based on local conditions of the pandemic. Therefore, by strategically modulating the time between dose administrations, local public health systems can improve the pandemic contention and dynamically address emergent challenges.

Moreover, this method enables vaccine manufacturers to quickly re-estimate the effectiveness of their vaccines against circulating variants, which can help detect evasive pathogen mutations. This analysis could help increase the utility of each dose, based on real-world information, without performing long and expensive clinical trials.

Concerning the limitations of this method, it is worth mentioning that it relies heavily on the availability of real-world data. Therefore, national or international databases with information about millions of individuals are required. Additionally, these databases must not have biases, as these perturbations to real-world data induce errors that could lead to the estimation of highly deviated vaccine effectiveness profiles. The final consideration may be a motivation for public health organizations to establish robust and trustworthy national databases not only for COVID-19, but for any other disease. In addition, although this study used the most general interaction model to extract the independent effect of each dose, the methodology can easily be adapted to consider more elaborate interactions or model the responses of vaccines designed against other diseases.

## 5. Conclusions

This paper introduces a novel approach for estimating the effectiveness profiles of vaccines. The method tackles the characterization problem by considering it as an optimization process, aiming to minimize the error and obtain the most accurate estimation of vaccine effects from the available data. Applying this approach makes it possible to independently estimate the effectiveness profiles of the first and second doses of two-dose vaccination schemes, offering valuable insights into their temporal dynamics and individual impacts. The individual evaluation of the effect of each dose enabled estimations of the time between doses that maintained the target effectiveness for the longest possible period, maximizing the protection obtainable with a pair of doses.

Several comparisons with previously available methods were performed, and qualitative agreement was found between the results of the proposed method and previously available information. However, such a comparison allows the appreciation of this novel approach’s remarkable precision and accuracy advantages, which are indispensable to evaluate the individual effect of each dose.

Finally, although this study focused on the effectiveness against symptomatic disease, the proposed strategy could easily be adapted to characterize time-dependent effectiveness profiles against hospitalization and death, which would offer new findings on these vaccines.

## Figures and Tables

**Table 1:** Minimum effectiveness values (E) required to promote convergence at different vaccination coverages (V) and basic reproduction numbers ($R_{0}$).

		Vaccination Coverage				
		60%	70%	80%	90%	100%
		Minimum Required Effectiveness Values, %				
$R_{0}$	1.1	16	13	12	11	10
	1.2	28	24	21	19	17
	1.5	56	48	42	38	34
	1.8	75	64	56	50	45
	2.1	88	75	66	59	53
	2.4	98	84	73	65	59
	3	---	96	84	75	67
	5	---	---	100	89	80
	8	---	---	---	98	88
	10	---	---	---	100	90

## Data Availability

The basic data used for performing this study are available in the links referenced in this article’s main text or the files included in its Supplementary Materials.

## Supplementary Materials

The following supporting information can be downloaded at: https://www.mdpi.com/article/10.3390/vaccines12010081/s1, Document SM1: Pseudocode of the tailored PSO algorithm used for the numerical deconvolution; Spreadsheet SM2: Spreadsheet with the optimization procedure for each dose decoupling; Spreadsheet SM3: Spreadsheet with the optimization procedure for each dose decoupling with an alternative interaction model; Video SM4a: Video containing an ordered sweep of each parameter of Equation (8); Video SM4b: Video containing a random exploration of the search space of the parameters of Equation (8); Document SM5: Table with the reference values and search intervals required for the fitting process of the individual vaccine effectiveness profiles; Document SM6: Details about the obtainment of Equation (12).
