# Supplementary material for: Mathematical Optimization Strategy for Effectiveness Profile Estimation in Two-Dose Vaccines and Its Use in Designing Improved Vaccination Strategies Focused on Pandemic Containment
Source: Vaccines (Basel). 2024 Jan 12;12(1):81. doi: 10.3390/vaccines12010081 (PMC10819090; doi:10.3390/vaccines12010081)
Supplement: Supplementary file 1 [file vaccines-12-00081-s001.zip › Supplementary Material SM1.pdf]

Supplementary Material SM1.

Tailored PSO algorithm used to fit the experimental data

Initialize the control parameters N, C_1, C_2, W_{min} , W_{Max}, V_{min} , V_{Max}, N_{iter}
Initialize the N particles

While iteration < N_{iter}
  For each particle
    Calculate the fitness
    Actualize the particle's optimal
    Actualize the population's optimal

    Actualize the particle's inertia

  For each particle
    Actualize the particle's velocity
    Actualize the particle's position
    Obtain a mean mobile of the particle's dimensions (for smoothness)
Return the population's optimal as the solution found
