# Supplementary material for: Mathematical Optimization Strategy for Effectiveness Profile Estimation in Two-Dose Vaccines and Its Use in Designing Improved Vaccination Strategies Focused on Pandemic Containment
Source: Vaccines (Basel). 2024 Jan 12;12(1):81. doi: 10.3390/vaccines12010081 (PMC10819090; doi:10.3390/vaccines12010081)
Supplement: Supplementary file 1 [file vaccines-12-00081-s001.zip › Supplementary Material SM5.pdf]

Supplementary Material SM5

Table S1. Reference values and search intervals for the fitting process of the individual vaccine effectiveness profiles.

Parameter	Reference values	Search interval
A_L	3.0	2.5 to 5.0
B_L	2.5	-2.0 to 4.0
C_L	0.02	-5.00 to 5.00
D_L	-70	-100 to -40
A_R	-5.0	-10.0 to -4.5
B_R	5.0	-5.0 to 5.4
C_R	0.015	0.010 to 5.000
D_R	50	0 to 100
Q	0.3	0.15 to 0.85
Δk	35	28 to 45
