# Supplementary material for: Mathematical Optimization Strategy for Effectiveness Profile Estimation in Two-Dose Vaccines and Its Use in Designing Improved Vaccination Strategies Focused on Pandemic Containment
Source: Vaccines (Basel). 2024 Jan 12;12(1):81. doi: 10.3390/vaccines12010081 (PMC10819090; doi:10.3390/vaccines12010081)
Supplement: Supplementary file 1 [file vaccines-12-00081-s001.zip › Supplementary Material SM6.pdf]

Practically speaking, the R_0 parameter implies how many individuals will get an infection for each active confirmed case. Therefore, in an unregulated system, the "apparent" basic reproductive number equals the basic reproductive number, as shown in Equation S1.

$$R_A = R_0 \tag{S1}$$

However, suppose the population under study is partially vaccinated (with a vaccine 100% effective). In that case, the "apparent" reproductive number will be artificially decreased, as some individuals that initially would get infected will now be protected by the vaccine. Such proportion vaccinated (P) will then weigh the R_0 parameter because only the susceptible individuals ($1 - P$) will now be implied in the process, as shown in Equation 2.

$$R_A = (1 - P)R_0 \tag{S2}$$

Then, suppose the population under study is partially vaccinated with a vaccine with an effectiveness lower than 100%. Then, the proportion vaccinated (P) needs to be weighted considering the expected value of protected individuals, that is, the product of the population vaccinated (P) and the expected effectiveness (E) as shown in Equation 3.

$$R_A = (1 - PE)R_0 \tag{S3}$$

Considering this work aims to induce convergence, the "apparent" basic reproductive number must be less than 1, as shown in Equation 4.

$$1 > R_A = (1 - PE)R_0 \tag{S4}$$

Finally, by algebraic manipulation, it is possible to obtain the required effectiveness to induce convergence in the system, considering that only a proportion of the population is vaccinated, as shown in the following expressions.

$$1 > (1 - PE)R_0 \tag{S5}$$

$$\frac{1}{R_0} > 1 - PE \tag{S6}$$

$$\frac{1}{R_0} + PE > 1 \tag{S7}$$

$$PE > 1 - \frac{1}{R_0} \tag{S8}$$

$$E > \frac{1}{P} - \frac{1}{PR_0} \tag{S9}$$
